# Adapting a trial design based on feasibility of recruitment where several treatment groups are possible and the outcome is long-term: pre-empt flexible-entry internal pilot study

**DOI:** 10.1186/1745-6215-16-S2-O18

**Published:** 2015-11-16

**Authors:** Lee Middleton, Jane Daniels, Kostas Tryposkiadis, Laura Gennard, Lisa Leighton, Siladitya Bhattacharya

**Affiliations:** 1 University of Birmingham, Birmingham, UK; 2 University of Aberdeen, Aberdeen, UK

## Background

Endometriosis occurs when the endometrium grows in abnormal locations outside of the womb, resulting in pain and reduced quality of life. See-and-treat surgery removes endometriotic lesions but the risk of recurrence is high. A HTA commissioning call asked to evaluate the long-term (three years) effectiveness of post-operative long-acting reversible contraceptives (LARCs) in preventing recurrence. A survey of gynaecologists indicated there was no consensus about which LARC (LNG-IUS or DMPA) or comparator (COCP or no treatment) should be evaluated. We designed a ‘flexible-entry’ internal pilot to assess whether a four-arm trial was feasible in light of possible strong patient preferences.

## Methods

During the pilot, patients could be randomised to two, three or four treatment options provided one was a LARC and one was a non-LARC. An assessment of feasibility based on recruitment to these options and a substantive trial design was considered by an independent oversight committee. This design was fixed to ensure adequate power at the end of the study.

## Results

The study ran for one year from April 2014 and 74 women were randomised. Only 5 (7%) women were happy to be randomised to all groups, with 60 (81%) having a LARC preference and 53 (72%) a non-LARC preference. Four-way and three-way designs were ruled out with a two-way (preferred LARC v COCP), stratified by LARC preference, considered feasible.

## Conclusions

Where multiple treatment options are available a flexible approach to randomisation in a pilot phase can be used to assess feasibility and adapt a trial design.

